# Enhanced Sunscreen Effects via Layer-By-Layer Self-Assembly of Chitosan/Sodium Alginate/Calcium Chloride/EHA

**DOI:** 10.3390/molecules27031148

**Published:** 2022-02-08

**Authors:** Chuntao Xu, Xuemin Zeng, Zujin Yang, Hongbing Ji

**Affiliations:** 1 School of Information Engineering, Zhongshan Polytechnic, Zhongshan 528400, China; chuntao@zspt.edu.cn; 2 School of Chemistry and Chemical Engineering, Guangxi University, Nanning 530004, China; 3 Fine Chemical Industry Research Institute, School of Chemistry, Sun Yat-Sen University, Guangzhou 510275, China; zengxm9@mail2.sysu.edu.cn; 4 School of Chemical Engineering and Technology, Sun Yat-Sen University, Zhuhai 519082, China

**Keywords:** layer-by-layer self-assembly, EHA (2-Ethylhexyl-4-dimethy-laminobenzoate), sustained release, sunscreen nanocapsule

## Abstract

The sunscreen nanocapsules were successfully synthesized by the way of layer-by-layer self-assembly using charged droplets (prepared by emulsification of LAD-30, Tween-80 and EHA (2-Ethylhexyl-4-dimethylaminobenzoate)) as templates. Chitosan/sodium alginate/calcium chloride were selected as wall materials to wrap EHA. The emulsions with the ratio of Tween-80 to EHA (1:1) were stable. A stable NEI negative emulsion can be obtained when the ratio of Tween-80 and LAD-30 was 9:1. Chitosan solutions (50 kDa, 0.25 mg/mL) and sodium alginate solutions (0.5 mg/mL) were selected to prepare nanocapsules. The nanocapsules were characterized via some physico-chemical methods. Based on the synergistic effects of the electrostatic interaction between wall materials and emulsifiers, EHA was effectively encapsulated. DLS and TEM showed that the sunscreen nanocapsules were dispersed in a spherical shape with nano-size, with the increasing number of assembly layers, the size increased from 155 nm (NEI) to 189 nm (NEII) to 201 nm (NEIII) and 205 nm after solidification. The release studies in vitro showed sustained release behavior of the nanocapsules were observed with the increase of the number of deposition layers, implying a good coating effect. The sunscreen nanocapsules could control less than 50% the release of EHA after crosslinking of calcium chloride and sodium alginate, which also could effectively avoid the stimulation of the sun protection agent on the skin.

## 1. Introduction

Recently, the skin damage caused by ultraviolet (UV) radiation has caused widespread concern. In particular, excessive exposure to UV radiation can bring some serious diseases, such as sunburn, photoageing and skin cancer [1]. Therefore, it is urgent to develop some efficient sunscreens to protect the skin from UV damage [2]. Generally speaking, sunscreens generally include inorganic and organic compounds. EHA (2-Ethylhexyl-4-dimethylaminobenzoate), as a UV filter, is widely used in sunscreens and cosmetics. However, the practical application of EHA has been limited due to its poor photostability. In addition, the strong permeability of EHA into the deep skin could adversely affect skin biology [3]. Therefore, some efficient and safe methods have been studied to protect our skin.

In order to solve these problems, much effort has been made to develop micro-nanocapsule technology for the sunscreen encapsulated [4]. It was a technology that could be used in the micro-nanosize composite, emulsification and construction techniques to encapsulate and release active materials under specific conditions [5]. Due to its nano-size, surface effect and core–shell structure, the nanocapsules have been used in many areas, such as food [6], medical [7], textile [8], cosmetic [9] and so on. EHA encapsulated by nanocapsules not only had a certain sunscreen effect, but also effectively avoided its direct contact with skin. However, good dispersibility and sustained sunscreen-release property have been two main challenges for the design of efficient nanocapsules. It has been reported that most of the sunscreen nanocapsules had poor dispersibility in emulsion [10], limiting their application in sunscreen products. The way of layer-by-layer self-assembly (LBL) was a new strategy to prepare the nanocapsules with nano-size and good dispersibility [11]. Due to its mechanism that was deposited oppositely charged polyelectrolytes with the interaction of electrostatic interaction, thus the morphology [12], size and other properties of the nanocapsules could be controlled by adjusted the number of assembly layers [13]. Unlike other methods, the nanocapsules prepared by LBL technology could be adjusted according to demands, such as controlled the diffusion rate by adjusted the thickness of wall, changed the shape by adjusted the pH and ionic strength of solutions, prepared multi component composite shell by used many kinds of materials [14]. However, it was necessary to prepare more than ten layers to construct the stable capsule [15]. The ionic emulsifier was used to emulsify sunscreen to prepare the charged droplets, and then adsorbed the wall on the surface of the droplet by layer self-assembly.

Chitosan (CS), is a natural biopolymer linear polysaccharide. Its chemical name is (1,4)-2-amino-2-deoxy-β-d-glucan. It is the deacetylation product of chitin. Chitosan is the only alkaline polysaccharide in nature. Its basic structural unit is glucosamine, which is a substance existing in the human body [16]. Chitosan has a good affinity with human cells, no toxicity, no stimulation, no rejection, good histocompatibility, and is safe in vivo. Under acidic conditions (pH < 5), chitosan can be swelled to form gels. The drug is released slowly, hydrophilic, but insoluble in water and stable in an alkaline medium [17]. Chitosan has positive electricity and good biological adhesion [18], which enhances its adhesion and prolongs the drug residence time under the condition of negative charge on the mucosal surface. Chitosan has strong plasticity and can be made into films, pressed into tablets, particles [19], microspheres and so on. Chitosan has many excellent physical, chemical, biological and pharmaceutical properties, which can not be compared with other sustained-release materials [20]. In addition, it has rich sources and low prices, so it is the best raw material for drug sustained-release materials.

Sodium alginate is a compound extracted from marine algae. A substance is defined as a natural polymer “generally considered safe” by the US Food and drug administration. Sodium alginate is a linear anionic polysaccharide compound whose molecular chain is composed of β-d-mannuronic acid and α-l-guluronic acid is polymerized by (1–4) glycosidic bond [21]. A large number of negatively charged carboxyl groups [22] in sodium alginate interact with the positively charged primary amino groups in chitosan to form composite microspheres. The composite microspheres can be used as the carrier of sunscreen and enhance the stability and embedding rate of sunscreen.

**Scheme 1:**
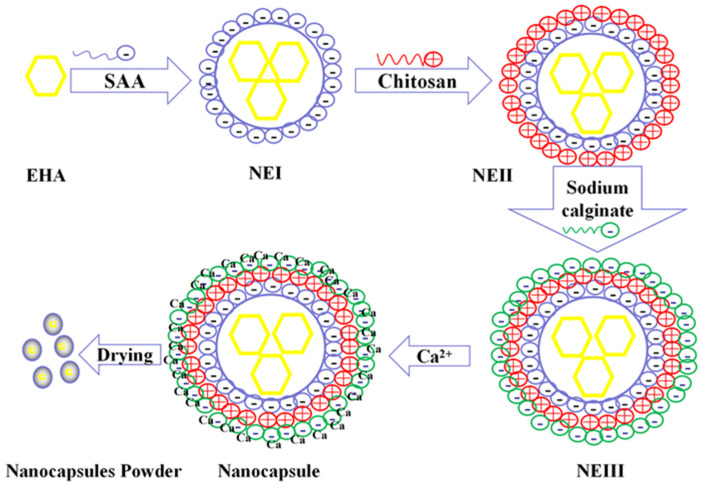
The synthetic route of the EHA nanocapsules by layer-by-layer self-assembly of chitosan/sodium alginate/calcium chloride/EHA.

The droplets with negative charge were prepared by emulsification of LAD-30, Tween-80 and EHA in this paper. Then, the sunscreen nanocapsules were obtained by self-assembly chitosan and sodium alginate on the surface of charged droplets, following solidified by calcium chloride in Scheme 1. The stability of nanocapsules were evaluated by measuring the size and zeta potential of dynamic light scattering (DLS). The release of EHA from the EHA nanocapsules were also investigated using dialysis method. Based on the emulsification and electrostatic incorporation the sunscreen nanocapsules showed good stability and sustained EHA release properties, which also showed an excellent sunscreen effect.

## 2. Materials and Methods

### 2.1. Materials and Reagents

Chitosan (Mn = 50,000) was obtained from Nantong Xingcheng Biologics Co., Ltd. (Jiangsu, China). 2-Ethylhexyl-4-dimethylaminobenzoate (EHA) was supplied from Shanghai Macklin Biochemical Co., Ltd. (Shanghai, China). Tween-80 was purchased from Tianjin Damao Chemical Reagents Co., Ltd. (Tianjin, China). Sodium alginate (ALG) was brought from Shenzhen Yinuo Food Ingredients Co., Ltd. (Shenzhen, China). and Sodium Lauroamp hoacetate (LAD-30) were acquired from Guangzhou Mingwang Biotechnology Co., Ltd. (Guangzhou, China). Other reagents were of analytical grade and used without further purification. 

### 2.2. Preparation of the Sunscreen Nanocapsules

#### 2.2.1. Preparation of Primary Nanoemulsions (NEI)

The ideal emulgators were chosen according to the previous literature [23]. A certain amount of emulgator and EHA were added into a beaker, which was immersed in a water bath at 30 °C. Then, 50 mL deionized water was added into the beaker and kept continue stirring for 15 min at the 750 rpm. Finally, a series of milky white emulsions with different concentrations of emulgator was obtained (5, 10, 15, 20 and 25 mg/mL). The absorbance of the distilled milky white emulsion was measured by a UV-Vis spectrophotometer (Shimadzu Corporation, Kyoto, Japan) at 540 nm. The mixed emulgators with different proportions were prepared according to the previous literature [24]. 1.0 g of the mixed solutions containing Tween-80, LAD-30 (Tween-80: LAD-30, 10:0, 10:1, 10:3, 10:5 and 10:7 at the whole mass, respectively) and 1.0 g of EHA were immersed into water bath at 30 °C under siting at 750 rpm. Then, 50 mL of deionized water was added into the beaker. After stirring for another 15 min, NEI were obtained and measured by UV-Vis at 540 nm.

#### 2.2.2. Preparation of NEII and NEIII Nanoemulsions

The NEII and NEIII nanoemulsions were prepared by layer by layer self-assembly [25]. 10 mL NEI and 10 mL chitosan solutions with different concentrations (0.1, 0.25, 0.5, 0.75, 1.0, 1.5, 2.0, and 2.5 mg/mL, respectively) and molecular weight (30, 50, and 100 kDa, respectively) were mixed in a beaker. After 15 min stirring, the NEII nanoemulsions were obtained. Then, 20 mL NEII nanoemulsions were slowly added to 20 mL of different conernations (0.25, 0.5, 1.0, 1.5, and 3.0 mg/mL, respectively) of sodium alginate solutions under stirring at 750 rpm. After 15 min, the NEIII nanoemulsions would be obtained.

#### 2.2.3. Preparation of Sunscreen Nanocapsules

20 mL of NEIII nanoemulsions was slowly added into a series of concentrations of calcium chloride solutions (2.0, 4.0, 6.0, 8.0 and 10.0 mg/mL, respectively). After stirring for 15 min at 750 rpm, the sunscreen nanocapsules would be obtained.

### 2.3. Characterization of EHA Nanocapsules

Dynamic light scattering (DLS) (Zetasizer Nano ZS90, Malvern Instruments, Worcestershire, UK) was used to measure the distribution and zeta potential of the EHA nanocapsules. A certain amount of EHA nanocapsules were added into the sample bottle and mixed with 5 mL deionized water and dilute to 5 μg/mL, shaken and mixed evenly, measured 1 mL with a pipette gun, placed in the sample cell, and tested the particle size and zeta potential at 25 °C. An average value was obtained by three repeated measurements at 25 °C for each sample. Transmission electron microscopy (TEM) was conducted on JEM-2100 (HR) electron microscope at an acceleration voltage of 200 kV. The samples were prepared by placing a drop of the solution of the EHA nanocapsules onto the lacey support films and dried at 30 °C for 10 h.

### 2.4. The Stability of the EHA Nanocapsules

The stability of the EHA nanocapsules was tested according to the variations of size distribution measured by DLS during 7 days. The size distributions of NEI, NEII and NEIII nanoemulsions were also measured by DLS, which showed the influence of preparation processes such as pH, the speed of stir, the concentrations of chitosan solutions on the size distribution.

### 2.5. Release of EHA from the EHA Nanocapsules In Vitro

The release of EHA from the EHA nanocapsules was also investigated using dialysis method [26]. A certain amount of nanocapsules solutions were diluted to 4 mL by phosphate-buffered solution (PBS) at pH 6.5. Then, the solutions were transferred to a dialysis tube (MWCO: 3500 Da) and followed dialyzed against 30 mL of the PBS at 37 °C. At a certain time interval, 4 mL of release media was sampled and replaced with an equal volume of fresh media. The amount of EHA was determined by HPLC. Chromatographic conditions: chromatographic column: wondasil c18-wr column (4.6 × 150 mm, 5 μm). The mobile phase was methanol: Water (92:8), The flow rate was 1.0 min/mL. The detection wavelength was 311 nm, Column temperature: 30 °C, and the injection volume was 10 uL. The cumulative releases were calculated as:

(1) $$Release(\%)=\frac{(V_{t}\sum_{0}^{n-1}C_{t}+V_{0}C_{0})\times 100}{m}$$

where *V*_0_, *C_0_*, *V_t_*, *C_t_* and *m* were the total volume of the solutions, the initial concentration of EHA, the volume sampled at *t*, the concentration of EHA at *t* and the total mass of EHA, respectively.

### 2.6. Evaluation of Cutaneous Transdermal Permeation of EHA Nanocapsules In Vitro

Seven-week-old male hairless mice were obtained from the Medical Laboratory Animal Center of Sun Yat-Sen University. The animal protocol was approved by the University of Sun Yat-Sen University Health Sciences Center Institutional Animal Care and Use Committee. Animals were sacrificed by CO_2_ asphyxiation and full-thickness abdominal and dorsal skin was excised. Any extraneous subcutaneous fat was removed from the dermal surface. The skin samples were stored at −18 °C until utilized. At the time of experimentation, skin samples were slowly thawed, cut into pieces for appropriate size, and mounted on standard Franz diffusion cells (TP-6, Tianguang Photoelectric Instrument Co., Tianjin, China). Each diffusion cell (donor surface area 2.25 cm^2^, receptor volume 10.1 mL) contained isotonic phosphate-buffered solution (pH 7.2). The receptor fluid was maintained at 37 ± 0.5 °C and continuously stirred at 400 rpm using magnetic stirring bars [27]. In order to investigate the transdermal absorption of EHA nanocapsules, EHA nanocapsules were added to the sunscreen formula to make EHA nanocapsules sunscreen. The addition concentration of EHA was 5%, compared with the unpacked EHA sunscreen. The sunscreen formulations were prepared according to the 2015 edition “cosmetic safety technical guide” (Table 1). Following a 1-h hydration period, 4.5 mg of EHA nanocapsules sunscreen formulation (the content of EHA was 100 μg·cm^−2^) was spread uniformly over each skin. After the start of the test, suck 2 mL of receiving solution at 0.5, 1, 2, 4, 6, 8, 10 and 12 h, respectively, and immediately replenish the same amount of receiving solution. The content of EHA (*C*_*n*_) in each receiving solution was determined by HPLC. The cumulative value was the calculated permeability according to the following formula (*Q*, μg·cm^−2^) as

(2) $$Q=(\overset{n}{\underset{i=1}{\sum }}C_{n}\times V)/A$$

where *C_n_* is the EHA concentration measured at the nth sampling point (μg·mL^−1^), *V* is the sampling volume (mL), *A* is the penetration area (cm^2^). Taking *Q* as the ordinate and time as the abscissa, the in vitro transdermal curve was obtained.

The diffusion cells were dismantled, and each sample was carefully cleaned by the application of a cotton swab imbibed with 1.0% (*w*/*w*) aqueous solution of sodium dodecyl sulfate in order to mice skin cleansing with liquid soap. EHA deposited in the skin was extracted by cutting the skin samples into small pieces and soaking in 4 mL of acetonitrile overnight with continuous stirring at room temperature. The extraction samples were centrifuged at 8000 rpm for 15 min and diluted (if required) prior to HPLC analysis.

### 2.7. The Release Kinetics Mode of the EHA Nanocapsules

The release behavior of the EHA nanocapsule was investigated by three kinds of kinetics mode such as zero-order kinetics model, first-order kinetics model and Hiauchi model to obtain the best kinetics mode. The *Q* represented the cumulative release of EHA and *t* was the release time of EHA from the EHA nanocapsule.

Zero-order kinetics model:

(3) Q=a+bt

First-order kinetics model:

(4) $$\ln(Q_{\infty }-Q_{t})=a+bt$$

Hiauchi model:

(5) $$Q_{t}=bt^{1/2}$$

### 2.8. UV Absorption Effect of the EHA Nanocapsules

According to the light industry standard QB/T2410-1998 “The evaluation method of sunscreen effect of sunscreen cosmetic-UV absorbance method”, the sunscreen performance of the EHA sunscreen nanocapsules was tested by UV-Vis. In detail, the same contents of EHA and EHA nanocapsules were smeared on the 3 M tape, and then they were scanned from 280 to 400 nm. Finally, the UV absorption curves were drawn based on the above results.

### 2.9. Statistical Analysis

The results were evaluated according to the Analysis of Variance (ANOVA), and the means were compared by the Tukey test, considering the significance level of 5% (*p* < 0.05), using the software STATISTICA 7.0 (StatSoft Inc., Tulsa, OK, USA).

## 3. Results and Discussion

### 3.1. Screening and Blending of Emulsifier

**Figure 1:**
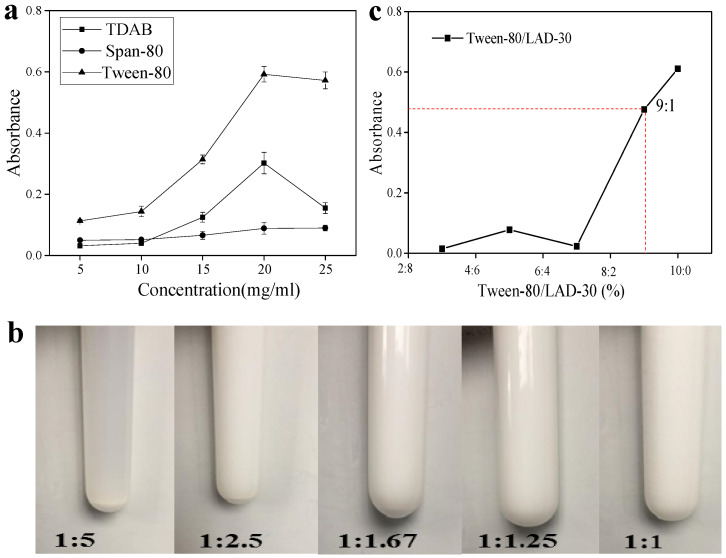
The influence of the stability of emulsion: (**a**) different emulsifiers, (**b**) the proportion of emulsifier and core material, (**c**) proportion of Tween/LAD-30.

According to the principle of emulsion drop birefringence, the absorbance of emulsifiers TDAB, Tween-80 and Span-80 was measured at 540 nm by UV-Vis, using EHA as core materials. As shown in Figure 1a, three kinds of emulsifiers presented the same trends, and the absorbance increased at the beginning, and then decreased with the increasing of concentration of emulsifiers. The stability of the emulsion in the emulsification process can mainly be controlled by reducing the interfacial tension of the oil/water interface, and it can form a boundary film [28]. When the concentration was 20 mg/mL, the maximum value of the absorbance was observed, indicating that the emulsion was the most stable under the concentration. Compared to TDAB and Span-80, the emulsion formed by Tween-80 showed the maximum absorption value of 0.592, implying that Tween-80 can emulsify EHA very well.

As we know, the steadiness of the emulsion was also affected by the concentration of core materials. A series of emulsions with different mass ratios of Tween-80 and EHA was shown in Figure 1b. The precipitation appeared in emulsions when the ratio of Tween-80 to EHA was less than 1:1 after a period of static storage, but there was no precipitation in the emulsion with the ratio of 1:1 (Tween-80: EHA), suggesting the emulsions with the ratio of Tween-80 to EHA (1:1) were stable. Therefore, the 1:1 molar ratio was used in the following experiment.

In order to obtain a better emulsification effect, blending emulsifier is a good method that has been widely investigated by researchers [29]. As shown in Figure 1c, the absorbance increased at the beginning, then slightly decreased and increased significantly with increasing the molar ratio of Tween-80 to LAD-30 from 2:8 to 9:1. The maximum UV absorption value was obtained when the ratio was 9:1, which indicated the emulsions prepared under this condition were most stable. It was due to the fact that the negative charge had an influence on the emulsifying effect [30]. When the content of LAD-30 was reduced from 3:7 to 5:5, the electrostatic repulsion between emulsifier molecules was reduced, which was beneficial to the surface arrangement of the EHA and the strength of the oil/water mask, thus improving the stability of the emulsion [31]. However, the electrostatic repulsion between droplets was also decreased with the decreasing the content of LAD-30, leading to the droplet colliding and rupturing during movement, decreasing the stability of the emulsion. When the ratio increased to 9:1, the emulsifier molecules with lower electrostatic repulsion among them, which could be arranged on the surface of the core, forming a high intensity oil/water mask and effectively preventing the collision and reunion between the droplets and increasing the stability of the emulsion. In order to prepare negatively charged droplets as self-assembled templates, the ratio of Tween-80/LAD-30 (9:1) was used to prepare the primary nanoemulsions.

### 3.2. Prepare of Sunscreen Nanocapsules

**Figure 2:**
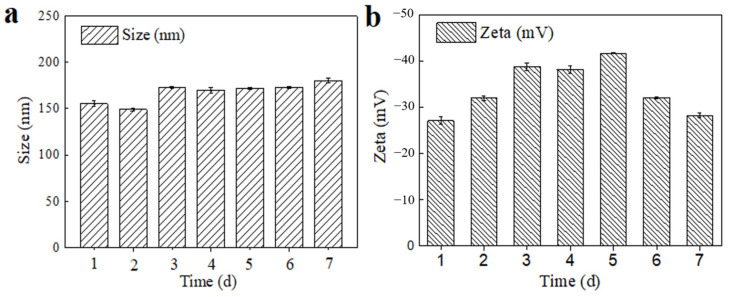
NEI of (**a**) size, (**b**) zeta on the stability.

In this study, sunscreen nanocapsules were successfully prepared by layer-by-layer self-assembly [32]. NEI with negative charge was prepared using Tween-80 and LAD-30 as emulgators. Then, NEII and NEIII nanoemulsions were obtained in the presence of chitosan solutions and sodium alginate solutions, respectively. Finally, with the solidification of calcium chloride solutions, the EHA nanocapsules were obtained. The effects of stirring speed, stirring time and EHA concentration on the stability of NEI were investigated by DLS. The results showed that the relatively stable primary emulsion could be achieved Under optimal process conditions, 750 pm, 12.5 min and 10 mg/mL of EHA. The stability of the obtained NEI was further investigated by DLS. Figure 2a showed the size of NEI tended to increase slightly and then be stable in 7 days, which was due to the hydration aquation of NEI. The size of NEI was 170 nm, indicating the low austenite ripening rate [33] and good NEI stability. The zeta potential of NEI was also measured by DLS. As shown in Figure 2b, NEI showed negative zeta potential with −27.1 mV, and zeta potential increased at the beginning. and then reduced to negative charges in 7 days, which was consistent with the results of particle size, indicating that NEI has good stability and could be used as a self-assembly template.

**Figure 3:**
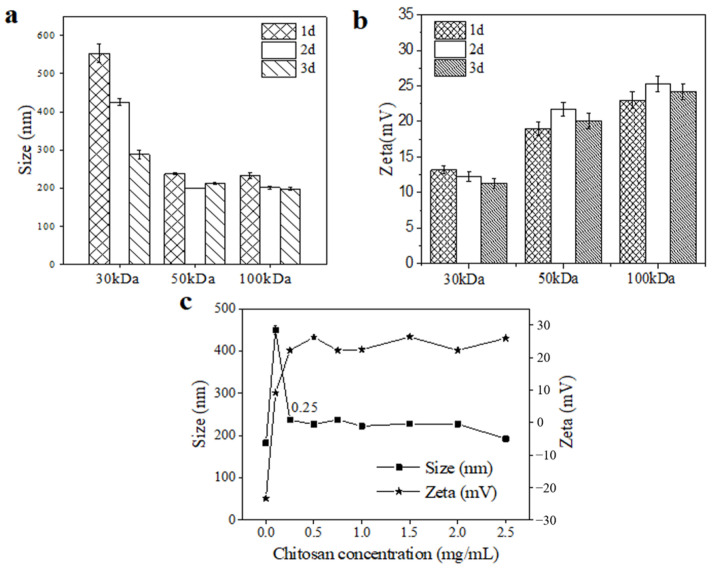
The effects of (**a**,**b**) molecular weight of Chitosan, (**c**) concentration of chitosanon the stability of NEII:.

It has been reported that the molecular weight and concentration of wall materials have an influence on the stability of emulsions prepared by layer-by-layer self-assembly [34]. Therefore, three kinds of NEII were prepared with a different molecular weight of chitosan (30 kDa, 50 kDa and 100 kDa, respectively) in this study. The size and potential were also measured by DLS. Compared to the size variation of NEII prepared by chitosan with 30 kDa, the negligible changes were shown in Figure 3a. For the size of NEII prepared by 50 kDa and 100 kDa chitosan in 3 days, it may due to the higher molecular weight of chitosan with more -NH_2_ groups, leading to higher positive zeta potential and reducing the absorption between NEII drops, which was confirmed by Figure 3b. Meanwhile, as shown in Figure 3b, the positive zeta potential was further demonstrated that NEII were successfully prepared by absorbing chitosan in the surface of NEI. The same results were observed in Figure 3c. The stability of NEII prepared by different concentrations of chitosan indicated that the concentrations of chitosan have significant effects on the stability of NEII. Then, 0.25 mg/mL chitosan solutions (50 kDa) were used to prepare NEII. Moreover, the conditions of preparing NEII were also investigated. Optimized process conditions such as pH = 4.5, 750 rpm and 15 min have also been obtained.

**Figure 4:**
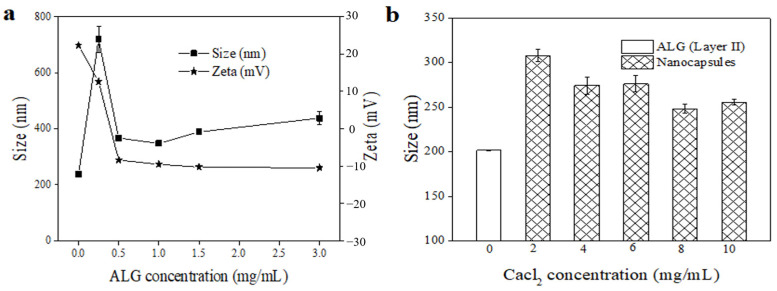
The effects of (**a**) concentration of sodium alginate, (**b**) concentration of CaCl_2_ on the stability of NEII.

The same way was used to obtain NEIII by absorbing the sodium alginate on the surface of NEII. The negative zeta potential shown in Figure 4a indicated the sodium alginate with a negative charge at pH = 4.5 was successfully absorbed on the surface of NEII and the NEIII was obtained. As the concentration of sodium alginate solutions increased, the size of NEIII increased and then reduced, and finally stabilized when the concentration of sodium alginate solutions was higher than 0.5 mg/mL. Figure 4b showed the size would be larger after the solidification by calcium chloride than NEIII, indicating the surfaces of NEIII were successfully solidified by calcium chloride and the sunscreen nanocapsules were obtained. The lower or the higher concentration of calcium chloride made the sunscreen nanocapsules lose or gather with bigger sizes [35], which was not beneficial for the stability and application of the EHA nanocapsules. The smaller particle size difference between the sunscreen nanocapsules and NEIII indicated the existence of solidification, which made the nanocapsules more compact and stable.

### 3.3. Characterization of Sunscreen Nanocapsules

**Figure 5:**
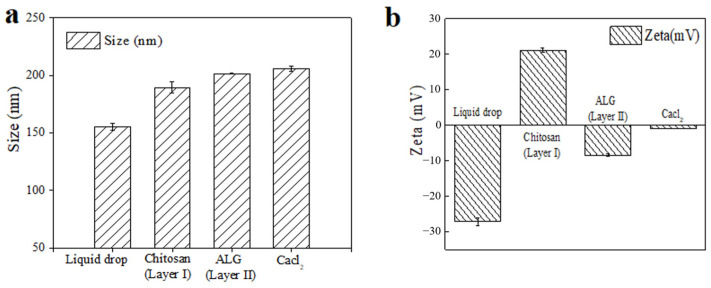
Change on droplet: (**a**) size, (**b**) zeta.

The size and zeta potential of the EHA nanocapsules were characterized by DLS and TEM. As shown in Figure 5 and Table 2, with the increasing number of assembly layers, the size increased from 155 nm (NEI) to 189 nm (NEII) to 201 nm (NEIII) and 205 nm after solidification; the zeta potential was changed from −27.1 mV to +21.1 mV, then to −8.44 mV, which indicated the successful assembly of each layer and the successful preparation of the nanocapsules [36]. The results indicated that the size and zeta potential of the EHA nanocapsules were 205 nm and −8.44 mV, respectively.

**Figure 6:**
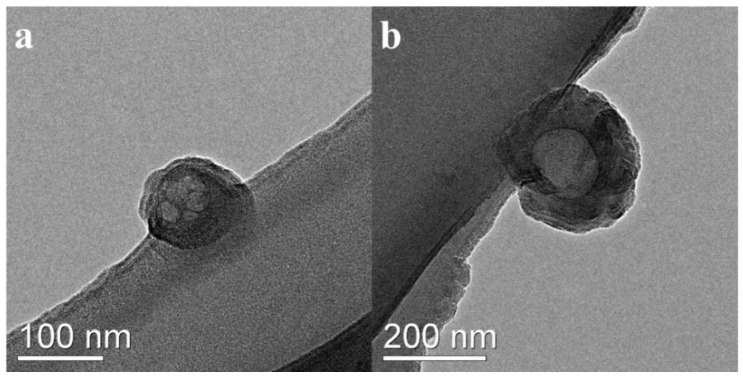
TEM image of (**a**) NEI and (**b**) nanocapsule.

TEM observation (Figure 6) also showed the NEI and the EHA nanocapsules dispersed in spherical shape with nano-size. The bright center and the gray surrounding indicated that NEI and the EHA nanocapsules had core–shell structure, which was due to the density difference between the center and the surrounding leading to the different electron beam scattering angle to the core and shell, causing the imaging brightness to be different [37]. The average diameter of sunscreen nanocapsules on the basis of the TEM image (200 nm) was larger than the size of NEI, which further indicated the sunscreen nanocapsules were successfully prepared.

### 3.4. The Release Behavior of EHA from the EHA Nanocapsules In Vitro

**Figure 7:**
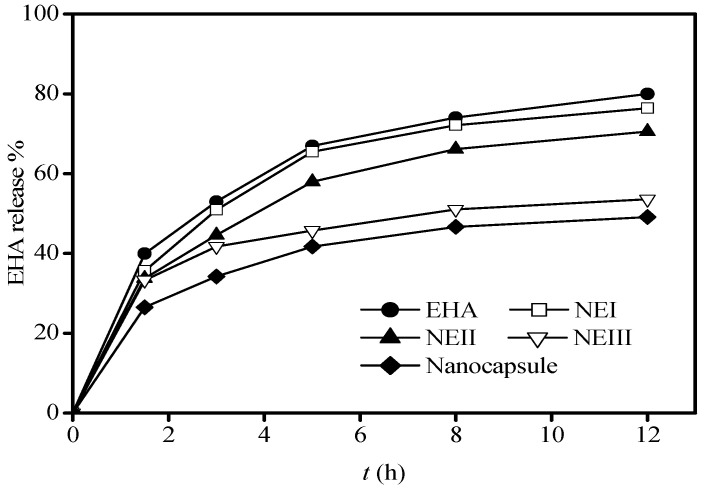
Cumulative release rate of EHA from sunscreen nanocapsules.

As an ideal sunscreen nanocapsule, zero and the controlled EHA-release performance are the main challenges [38]. Therefore, the EHA release behavior of the EHA nanocapsules was investigated in the acetate buffer solution (ABS, pH 6.5), compared with free EHA, NEI, NEII and NEIII. The cumulative amount of EHA release was monitored from the EHA nanocapsules solutions placed in a dialysis tube. As shown in Figure 7, the free EHA showed the highest release rate of EHA among emulsions and nanocapsules in the same period. As the layer increased, the release rate of EHA was reduced. About 80.0%, 76.4%, 70.5% of EHA could be released from free EHA, NEI and NEII, respectively, while less than 50% was released from sunscreen nanocapsules, and no burst release was observed. The adding of the layer would delay the EHA release and display more sustained EHA release behavior [39]. EHA release depended on the number of layers indicated that sunscreen nanocapsules with higher layer showed a lower release rate of EHA than free EHA and NHI, NHII, NHIII with no or lower layer in the same time period. It has been reported that EHA is released by diffusion [40]. The absorbing of chitosan with high molecular weight on the surface of NEI made the release rate from NEII slower than NEI. Similarly, the release rate of EHA from NEIII with sodium alginate outside was slower than NEII. Due to the cross-linking between calcium ion and alginate outside on the surface of NEIII, the sunscreen nanocapsules showed the slowest release rate of EHA, which effectively avoids the irritation of EHA to skin. These results confirmed that the ideal sunscreen nanocapsules could be obtained by the way of layer-by-layer self-assembly.

### 3.5. The EHA Nanocapsules Transdermal Permeation In Vitro

**Figure 8:**
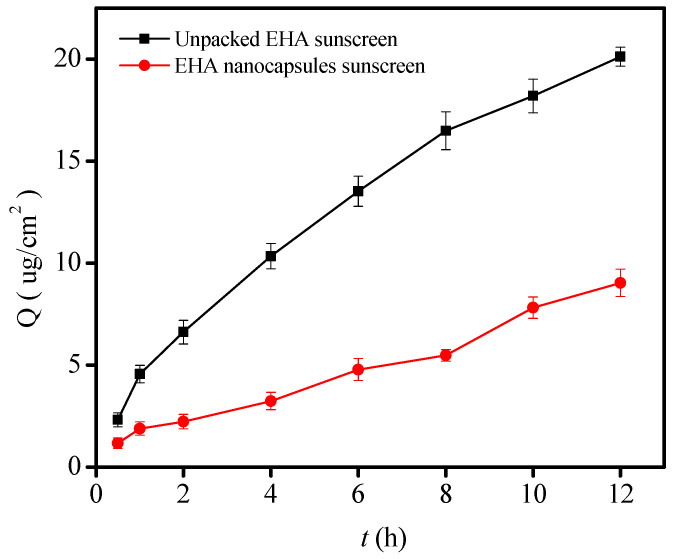
Transdermal accumulation of encapsulated and unwrapped EHA.

**Figure 9:**
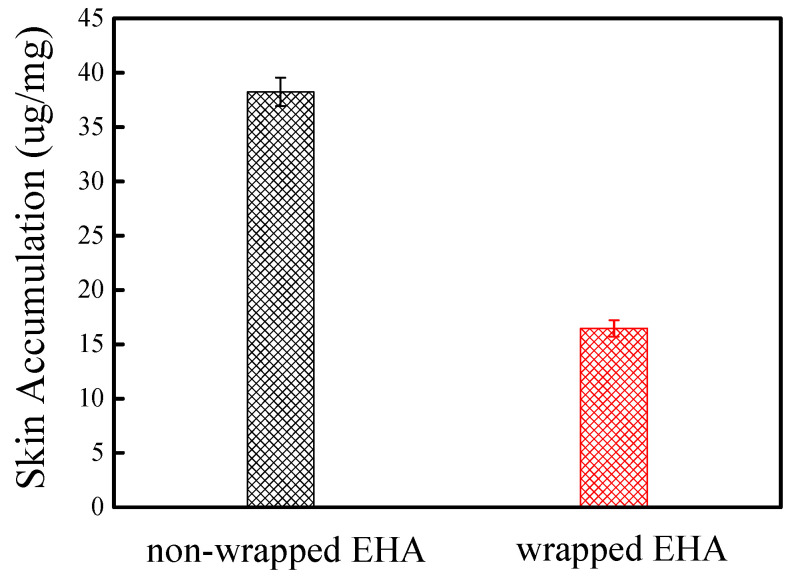
Skin accumulation of EHA.

In this experiment, sunscreen containing non-wrapped UV filters of EHA and the sunscreen with the EHA nanocapsules were used. The optimal exposure time of 12 h was selected according to the results of the previous study [41]. In order to compare the permeability of encapsulated sunscreen EHA and unpacked sunscreen EHA on the skin. The same sunscreen formula was added with wrapped and unwrapped EHA, respectively, and the concentration of EHA is 5%. The application concentration of the experimental skin was 100 μg·cm^−2^. The permeation results showed that a UV filter was present in the receptor compartment. The experimental results were shown in Figure 8, the cumulative permeability of non-wrapped EHA sunscreen within 12 h was 20.12 ± 0.47 μg·cm^−2^ and the EHA nanocapsules sunscreen was 9.03 ± 0.67 μg·cm^−2^. However, the cumulative permeability of non-wrapped EHA sunscreen (13.52 ± 0.74 μg·cm^−2^) was more than the EHA nanocapsules sunscreen (4.78 ± 0.54 μg·cm^−2^) within 6 h. Skin accumulation of EHA was found in Figure 9, the non-wrapped EHA (38.24 ± 1.32 μg·mg^−1^) within 12 h was higher than wrapped EHA (16.47 ± 0.76 μg·mg^−1^). Experiments showed that encapsulated EHA can greatly reduce transdermal absorption.

### 3.6. The Release Kinetics Model of Sunscreen Nanocapsules

**Figure 10:**
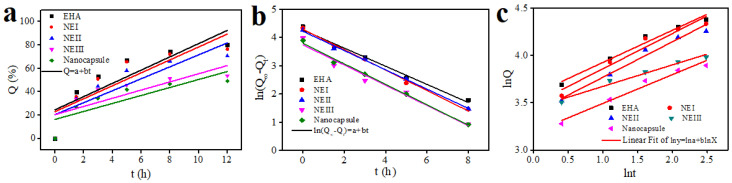
Release kinetics models of EHA from the EHA nanocapsules, (**a**) Zero-order kinetics Models, (**b**) First-order kinetics Models, (**c**) Higuchi kinetics Models.

The release behavior of EHA from sunscreen nanocapsules was matched by three kinds of model, zero-order kinetics model, first-order kinetics model and the Hiauchi model, with the comparison of free EHA, NEI, NEII and NEIII. The fitting curves were shown in Figure 10. The correlation coefficient *R* values were obtained by the equations and shown in Table 3. The lower *R* value calculated by zero-order kinetics equation indicated no burst release appeared during the EHA release for 12 h. The highest *R* value calculated by first-order kinetics equation indicated the release behavior of EHA was suitable for first-order kinetic equation [42]. The release behavior of EHA was a relatively stable process controlled by many mechanisms. The diffusion rate of EHA from the wall to the capsule was the key of the whole release behavior, similar to the mass transfer model [43], which was affected by the densification of membrane layer, the better densification leading to the slower release rate.

### 3.7. The UV Absorption Effect of Sunscreen Nanocapsules

**Figure 11:**
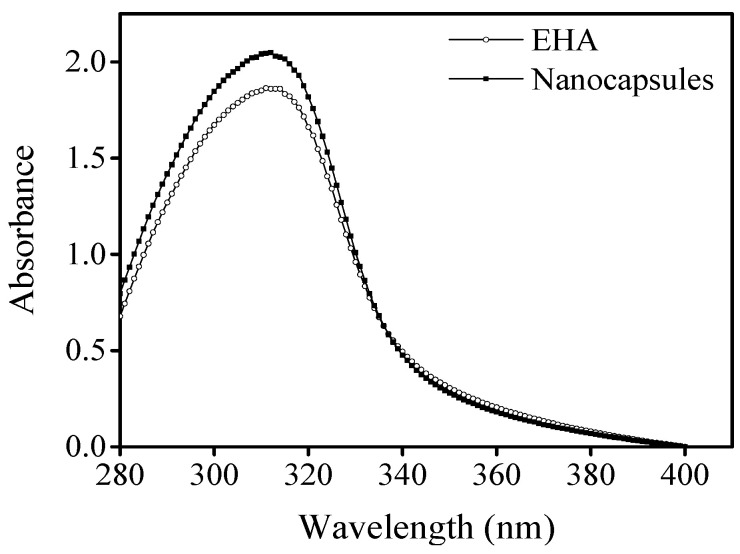
UV absorbability of the EHA nanocapsules.

In this study, the sunscreen effect of the EHA nanocapsules was investigated by UV-Vis. As shown in Figure 11, the free EHA and sunscreen nanocapsules all have high absorbance between 280 and 320 nm, and the maximum absorption intensity of 2.11 was 311 nm, which suggested sunscreen nanocapsules had a better sunscreen effect than free EHA. The maximum absorbance of the sunscreen nanocapsules was larger than that of free EHA, indicating that the sunscreen effect of sunscreen nanocapsules was better than that of free EHA, which due to the sunscreen nanocapsules were not only had the UV absorption function of chemical sunscreen, but also had the effect of physical sunscreen on ultraviolet reflection [44]. In detail, firstly, the EHA encapsulated by nanocapsules still had a good effect in sunscreen, secondly, the sunscreen nanocapsules as a spherical particle with nano-size had different degrees of scattering and refraction to ultraviolet rays except absorbing ultraviolet rays, leading to higher absorbance, thirdly, due to the nano-size, the sunscreen nanocapsules could be arranged closely on the 3 m tape and the clearance between particles was small, which had a certain diffraction effect on ultraviolet light, leading to the light transmittance reduced and the absorbency improved. In a word, all the results suggested the EHA encapsulated by nanocapsules were not only reduced the sunscreen effect, but also improved the sunscreen effect.

## 4. Conclusions

In this study, the sunscreen nanocapsules encapsulated by EHA were prepared successfully by layer-by-layer self-assembly. DLS indicated the nano-size and good stability of the sunscreen nanocapsules, and also showed the size of nanocapsules increased with the increasing of the layer. The zeta potential indicated that the sunscreen nanocapsules with negative charge and charge conversion would appear after each layer self-assembly. The sunscreen nanocapsules showed more sustained EHA release behavior than free EHA without an initial burst, and effectively delayed the EHA release. The UV absorption suggested the sunscreen effect of sunscreen nanocapsules, as well as free EHA and the sunscreen nanocapsules, were even higher than free EHA. The results implied the sunscreen nanocapsules prepared by the way of layer-by-layer self-assembly would have good potential used in sunscreen products with sustained release and high sunscreen effect properties.

## Figures and Tables

**Table 1:** Sunscreen formula tested in the experiments.

Components	Mass Fraction (%)
Phase A	
Cetearyl Alcohol	2.21
PEG-40 Castor oil	0.63
Sodium Cetearyl Sulphate	0.32
Decyl Oleate	15.00
2-Ethylhexyl-4-dimethylaminobenzoate (EHA)	5.00
Propylparaben /Methylparaben	0.40
Water	54.75
Phase B	
Disodium EDTA	0.10
Water	20.00
Carbomer	0.30
Sodium Hydroxide (45% solution)	0.30

**Table 2:** The incorporation parameters of EHA for the different nanocapsules prepared.

Stages	NEI	NEII	NEIII	Nanocapsule
*C* (EHA)/mg·mL^−1^	10	10	10	10
*C*(Tween-80)/mg·mL^−1^	10	10	10	10
Tween-80/LAD-30	9:1	9:1	9:1	9:1
*M*(CS)/KD		50	50	50
*C*(CS)/mg·mL^−1^		0.25	0.25	0.25
*C*(ALG)/mg·mL^−1^			0.5	0.5
*C*(CaCl_2_)/mg·mL^−1^				8.0
pH	10.0	4.5	9.5	7.0
Stirring speed (r/min)	750	750	750	750
Stirring time/min	12.5	12.5	12.5	12.5

LAD-30: Lauroamp hoacetate, CS: Chitosan, ALG: alginate.

**Table 3:** Related parameters of release kinetics model.

Models	Parameters				
	EHA	NEI	NEII	NEIII	Sunscreen Nanocapsules
Zero-order	0.6678	0.6708	0.7119	0.5269	0.6249
First-order	0.9875	0.9956	0.9935	0.9614	0.9927
Higuchi	0.9626	0.9296	0.9696	0.9668	0.9626

## Data Availability

Not applicable.

## Sample Availability

Samples of the compounds sunscreen nanocapsules are available from the authors.
